# Surgical Outcomes of Revision Endoscopic Dacryocystorhinostomy with or Without Concomitant Septoplasty: A Retrospective Comparative Study

**DOI:** 10.3390/medicina62071258

**Published:** 2026-06-30

**Authors:** Wook Hyun Jung, Ji Ho Choi, Sun Young Jang

**Affiliations:** 1 Department of Ophthalmology, Soonchunhyang University Bucheon Hospital, Soonchunhyang University College of Medicine, 170, Jomaru-ro, Bucheon 14584, Republic of Korea; slayernada77@naver.com; 2 Department of Otorhinolaryngology-Head and Neck Surgery, Soonchunhyang University Bucheon Hospital, Soonchunhyang University College of Medicine, 170, Jomaru-ro, Bucheon 14584, Republic of Korea; handsomemd@hanmail.net

**Keywords:** dacryocystorhinostomy, lacrimal duct obstruction, lacrimal duct, nasal septum

## Abstract

*Background and Objectives:* The combination of endoscopic dacryocystorhinostomy (En-DCR) and septoplasty enhances the surgical field, facilitating more effective treatment within the nasal cavity. This study compares the outcomes of revision En-DCR performed with or without concomitant septoplasty in patients with recurrent symptoms after primary En-DCR. *Materials and Methods:* A retrospective analysis was conducted on patients who underwent revision En-DCR after failure of primary En-DCR at our institution between March 2013 and June 2023. Patients were categorized into two groups: the revision En-DCR + septoplasty group and the revision En-DCR only group. Demographic information, intraoperative findings, and postoperative outcomes were thoroughly examined. *Results:* A total of 489 primary En-DCRs were performed on 381 patients, of whom 70.2% were female, with a mean age of 60.18 ± 14.38 years. Thirty-six patients (42 eyes, 8.6%) underwent revision En-DCR due to failed initial procedures, involving 30 unilateral and 6 bilateral cases. A total of 15 cases (35.7%) underwent combined endoscopic surgery with septoplasty, while 27 cases (64.3%) underwent revision En-DCR alone. Anatomical obstruction recurrences were observed in 1 case (6.7%) in the En-DCR + septoplasty group, whereas 6 cases (22.2%) experienced recurrence in the En-DCR-only group. The surgical success was numerically higher in the En-DCR + septoplasty group than in the En-DCR-only group (93.3% vs. 77.8%, *p* = 0.390). *Conclusions:* In this retrospective series, the combination of revision En-DCR with concomitant septoplasty yielded acceptable surgical outcomes despite more unfavorable nasal anatomy. This suggests that concomitant septoplasty can play a supportive role in revision cases by improving intranasal anatomy for anatomically challenging cases. However, because septoplasty was preferentially performed and the sample size was small, this study does not establish the independent benefit of septoplasty in revision En-DCR.

## 1. Introduction

Dacryocystorhinostomy (DCR) is a surgical procedure used to treat nasolacrimal duct (NLD) obstruction. DCR can be performed using two main approaches: external DCR and endoscopic DCR (En-DCR). En-DCR has gained widespread acceptance because it is less invasive, provides superior visualization of the lacrimal sac, offers better cosmetic outcomes, and avoids an external skin incision [1,2,3].

Despite significant advances in surgical techniques, DCR failure still occurs, and revision surgery remains challenging because of altered anatomy, scar formation, and a higher risk of recurrent obstruction. Previous studies have identified inadequate ostium size, insufficient sac marsupialization, cicatricial closure of the ostium, and granuloma formation as common causes of DCR failure [3,4]. In revision cases, membranous obstruction, cicatricial closure, granulation tissue, synechiae, and inadequate bony ostium have similarly been reported as major causes of failure [5,6].

In addition to these factors, anatomical variations within the nasal cavity may increase the technical difficulty of En-DCR [7]. Narrow nasal passages, particularly those caused by nasal septal deviation, may compromise endoscopic visualization and restrict surgical access to the middle meatus and lacrimal sac area [8,9]. Limited surgical exposure may contribute to incomplete rhinostomy formation and make postoperative management more challenging, potentially contributing to surgical failure [5,10]. Consequently, concomitant septoplasty has been proposed as a means of improving surgical exposure in selected patients with unfavorable intranasal anatomy.

Several studies have reported favorable outcomes when septoplasty was performed concomitantly with primary En-DCR, with reported success rates ranging from 88% to 95.8% [9,11]. In addition, concomitant septoplasty may facilitate endoscopic access and improve surgical exposure during the procedure [9,11,12]. However, whether septoplasty itself improves the anatomical or functional success of En-DCR remains controversial. Koval et al. found no significant differences in anatomical or functional success rates between patients undergoing En-DCR with septoplasty and those undergoing En-DCR alone [9]. Similarly, previous studies have reported that nasal cavity abnormalities do not significantly affect the success rate of primary En-DCR, even when some abnormalities are left untreated [13]. Therefore, although a narrow nasal cavity may increase surgical difficulty, its direct contribution to surgical failure remains uncertain.

Furthermore, data regarding the role of concomitant septoplasty in revision En-DCR remains limited. In our clinical practice, concomitant septoplasty is selectively considered in patients who experience recurrent obstruction after primary En-DCR and are found to have marked septal deviation or a narrow nasal cavity that may compromise endoscopic access to the previous rhinostomy site. These patients often represent cases in which surgical exposure was subjectively considered limited during the primary procedure. However, it remains unclear whether concomitant septoplasty influences surgical outcomes in this revision setting.

Therefore, the present study aims to compare the outcomes of revision En-DCR performed with or without concomitant septoplasty and to characterize the clinical features of patients who required concomitant septoplasty.

## 2. Materials and Methods

This retrospective study included patients who underwent revision En-DCR for persistent or recurrent symptoms after primary En-DCR performed at our institution between March 2013 and June 2023. Patients who had previously undergone lacrimal surgery at another institution, were lost to follow-up before 6 months postoperatively, or underwent En-DCR for lacrimal mass excision or biopsy were excluded.

Patients who experienced persistent or recurrent symptoms more than 3 months after primary En-DCR were evaluated for possible revision surgery. Revision En-DCR was considered only in patients with persistent epiphora despite appropriate conservative management and objective evidence of anatomical failure on nasal endoscopy, lacrimal irrigation, and/or probing. Concomitant septoplasty was not routinely performed in all revision cases. It was selectively undertaken in patients with marked septal deviation or a narrow nasal cavity that limited endoscopic visualization or surgical access to the middle meatus. Because this was a retrospective study, the degree of septal deviation was not graded using a standardized classification system. The indication for septoplasty was based on the joint assessment of the oculoplastic surgeon (SYJ) and the otorhinolaryngologic surgeon (JHC), each with more than 15 years of experience in their respective fields, when septal deviation or narrowing of the nasal cavity was considered sufficient to interfere with visualization, instrumentation, or access to the middle meatus and previous rhinostomy site.

Subjects were categorized into two groups: the revision En-DCR + septoplasty group and the revision En-DCR-only group. All primary En-DCR procedures were performed by a single surgeon (SYJ) under general anesthesia. Since a narrow nasal cavity or septal deviation alone was not considered an absolute indication for concomitant septoplasty [14], septoplasty was not routinely performed during primary En-DCR.

After induction of general anesthesia, the face was prepared with 10% povidone–iodine and draped. Both nasal cavities were packed with gauze soaked in epinephrine for vasoconstriction. The upper and lower puncta were enlarged with Vannas scissors. After removal of the nasal packing, a rigid endoscope was introduced into the ipsilateral nasal cavity, and an endoilluminator was inserted through the lower canaliculus to localize the lacrimal sac. Once the sac was identified, the overlying nasal mucosa and bone were removed with a drill and ethmoidal forceps to create an adequate bony ostium. The medial wall of the lacrimal sac was exposed, and probing of the upper and lower canaliculi from the nasal side was performed. Patency of the NLD was confirmed by lacrimal irrigation, after which bicanalicular silicone tube intubation was performed, and the tube was secured outside the nasal cavity. Nasopore was then packed in the nasal cavity for hemostasis.

In cases requiring concomitant septoplasty, septoplasty was performed first to improve surgical access. All of the concomitant septoplasty was performed by one otorhinolaryngologist (JHC) to correct the deviated septum and widen the operative nasal corridor. Revision En-DCR was performed with endoscopic identification of the previous rhinostomy site, removal of obstructing scar tissue or granulation tissue when present, enlargement or revision of the bony ostium as needed, confirmation of lacrimal system patency by probing and irrigation, and bicanalicular silicone tube intubation. Silicone tubes were routinely removed approximately 2–3 months after revision surgery, unless earlier removal was required because of tube-related irritation, displacement, infection, or other clinical reasons.

Data were extracted from the medical records, including demographic data, ophthalmic evaluation before surgery, dacryocystography (DCG) results, intraoperative findings, and postoperative records.

Follow-up visits were conducted at one day, one week, one month, two months, and six months, with additional visits as clinically indicated. Surgical success was defined as the absence of postoperative symptoms with the absence of NLD system blockage following surgery, as assessed by endoscopic examination and lacrimal irrigation.

This was a retrospective observational study in which all procedures were performed according to the tenets of the Helsinki Declaration. The study protocol was approved by the Institutional Review Board (IRB No. 2026-01-014). The board waived the need for informed consent because of the retrospective properties of this study.

Data are expressed as the mean ± standard deviation. Statistical analyses were performed using SPSS statistical software for Windows (version 27.0; SPSS Inc., Chicago, IL, USA). To compare clinical parameters of subjects, Fisher’s exact test, χ^2^ test, independent sample *t*-test, Fisher–Freeman–Halton exact test, and Mann–Whitney U test were conducted. A *p*-value of less than 0.05 was considered statistically significant.

## 3. Results

This study retrospectively analyzed 489 primary En-DCR cases (381 patients). Of these, 267 patients (70.1%) were female, and 114 (29.9%) were male. The mean age was 60.18 ± 14.38 years (range, 13–91 years). Among the 489 primary En-DCR cases, 253 (51.7%) were performed on the right side and 236 (48.3%) on the left side. The most commonly reported symptom was epiphora, followed by discharge and dacryocystitis. The baseline demographics and characteristics of all primary DCR cases are summarized in Table 1.

A total of 42 revision En-DCR cases in 36 patients met the inclusion criteria for this analysis. Among 42 revision En-DCR cases, 15 received revision En-DCR with septoplasty, and 27 received revision En-DCR alone. There were no statistically significant differences between the two groups in sex, age, or the operated side. General anesthesia was more frequently used in the revision En-DCR + septoplasty group, mainly because concomitant septoplasty required a wider surgical field and greater nasal manipulation. In contrast, local anesthesia was selected in some revision En-DCR-only cases when the nasal cavity was sufficiently accessible on preoperative or outpatient endoscopic examination, and the patient was considered able to tolerate the procedure. All of these patients underwent DCG before the first DCR, and in both groups, NLD obstruction was the most frequently observed finding in both groups. (14 cases in the revision En-DCR group and 5 cases in the revision En-DCR + septoplasty group). The demographics and characteristics of patients who received revision En-DCR are summarized in Table 2.

Intraoperative findings at revision En-DCR were also analyzed. The most common finding was a narrow nasal cavity, which was more frequently observed in the revision En-DCR + septoplasty group than in the revision En-DCR-only group (93.3% vs. 40.7%, *p* = 0.001). There were no significant differences between the groups in intraoperative bleeding, common canalicular obstruction, or small bony opening (Table 3).

Failure-related findings after primary En-DCR were additionally reviewed in the revision cohort and summarized in Table 3. To minimize overlap among postoperative findings, these were categorized as ostium obstruction/stenosis, scar or granulation tissue, and synechiae/cicatricial adhesion. Multiple findings could be recorded in the same case. Ostium obstruction/stenosis was observed in 24 cases (88.9%) in the revision En-DCR-only group and 14 cases (93.3%) in the revision En-DCR + septoplasty group (*p* = 1.000). Scar or granulation tissue was observed in 11 cases (40.7%) and 8 cases (53.3%), respectively (*p* = 0.525). Synechiae/cicatricial adhesion was significantly more frequent in the revision En-DCR + septoplasty group than in the revision En-DCR-only group (10 cases [66.7%] vs. 8 cases [29.6%], *p* = 0.027) (Table 3).

**Figure 1:**
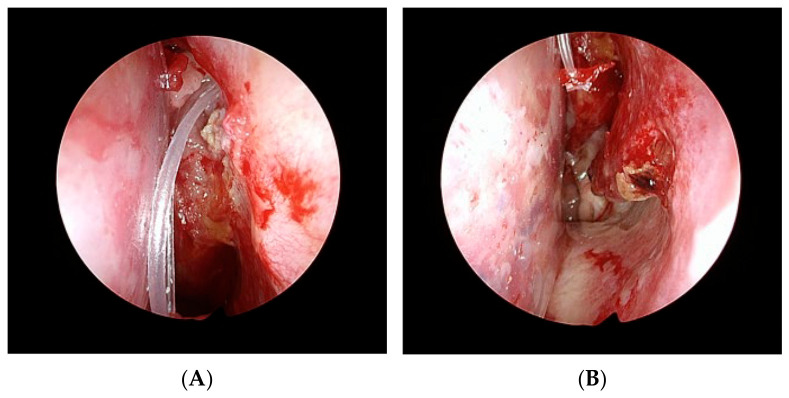
Representative endoscopic findings in a patient who received endoscopic dacryocystorhinostomy (En-DCR) combined with nasal septoplasty. (**A**,**B**) 56-year-old female patient underwent a primary dacryocystorhinostomy (DCR) on 24 August 2020. However, she presented epiphora after primary DCR. She had revision En-DCR with septoplasty on 13 December 2021. An intranasal photo shows the nasal cavity after revision En-DCR with septoplasty, with a silicone tube visible in the nasal cavity.

After revision surgery, the recurrence rate was numerically lower in the revision En-DCR + septoplasty group than in the revision En-DCR-only group (6.7% vs. 22.2%), although this difference did not reach statistical significance (*p* = 0.390) (Table 4). Postoperative complications were reviewed from the medical records. No major septoplasty-related complications, such as septal hematoma, septal perforation, uncontrolled nasal bleeding, or infection requiring additional intervention, were documented in the revision En-DCR + septoplasty group. Minor postoperative nasal crusting or self-limited bleeding was managed conservatively. In the revision En-DCR group, there were six subjects who experienced recurrence of symptoms. Among them, three subjects underwent revision En-DCR again, with one undergoing septoplasty concurrently. In the revision En-DCR + septoplasty group, one subject reported recurrence of symptoms after revision surgery and exhibited a narrow nasal cavity due to nasal septal deviation (Figure 1). The patient underwent removal of a fungus ball in the maxillary sinus, and a silicone tube was inserted during revision surgery. The subject underwent conjunctivoDCR four months after the revision-DCR procedure because of recurrence of symptoms and experienced complete resolution of symptoms.

## 4. Discussion

In our study, concomitant septoplasty was selectively performed in revision En-DCR cases with unfavorable nasal anatomy, particularly a narrow nasal cavity. Although the recurrence rate was numerically lower in the septoplasty group, this difference did not reach statistical significance. Nevertheless, the study remains clinically relevant because the septoplasty group included patients with more challenging nasal anatomy and showed acceptable postoperative outcomes. This suggests that concomitant septoplasty may improve surgical access in selected revision cases, although our study was not large enough to demonstrate a statistically significant difference in success rates.

Some patients in the septoplasty group may have had unfavorable nasal anatomy at the time of primary En-DCR. However, a narrow nasal cavity or septal deviation does not inevitably result in primary En-DCR failure, and septoplasty was not routinely performed during primary En-DCR solely on the basis of intranasal anatomical narrowing. Previous studies have reported that concomitant septoplasty during primary En-DCR does not necessarily improve anatomical or functional success, although it may facilitate surgical access or reduce intranasal complications [9]. Therefore, in the present study, concomitant septoplasty was selectively considered during revision En-DCR after additional otorhinolaryngologic evaluation when recurrent symptoms were accompanied by intranasal anatomical narrowing that could limit access to the previous rhinostomy site.

As mentioned above, reported success rates of primary En-DCR with septoplasty range from 88% to 95.8% [9,11]. The success rate observed in the revision En-DCR with septoplasty group in the present study (93.3%) fell within this previously reported range. However, direct comparison should be interpreted cautiously because of differences in clinical setting, patient population, and the limited sample size. Therefore, our findings should not be interpreted as evidence of equivalence between revision and primary procedures, but rather as demonstrating that acceptable outcomes can be achieved in selected revision cases requiring concomitant septoplasty.

It is well-established that a narrow nasal cavity is a significant factor in patients with NLD obstruction [15,16]. While a narrow cavity does not directly cause NLD obstruction, it can compromise the surgeon’s visibility and maneuverability during the procedure [15], potentially leading to primary En-DCR failure. In our study of revision En-DCR cases, a narrow nasal cavity was observed in 25 cases (59.5%). This high prevalence suggests that concurrently addressing the narrow nasal cavity with septoplasty may be particularly helpful for patients who experience recurrence following primary En-DCR.

Also, synechiae or granulations in the osteostomy site can cause failure of primary En-DCR [3,4]. The benefit of septoplasty with revision En-DCR is not limited to enhancing the surgical field. It also enlarges the middle meatus anatomically, which contributes to optimizing air circulation and reducing the risk of synechiae formation. In revision En-DCR with septoplasty cases, there were relatively more patients with narrow nasal cavities (93.3% vs. 40.7%, *p* = 0.001) but with lower rates of recurrence (6.7% vs. 22.2%, *p* = 0.390) than in only revision En-DCR cases. This suggests that septoplasty may help address unfavorable intranasal anatomy and improve success rates by establishing a stable nasal cavity in selected revision cases.

Some patients in the revision En-DCR-only group also had a narrow nasal cavity but did not undergo concomitant septoplasty. In these patients, uncorrected intranasal narrowing may have limited surgical access and contributed to recurrence. However, the effect of nasal septal deviation or septoplasty on DCR outcomes remains controversial. While some studies have reported no significant benefit of concomitant septoplasty on En-DCR success, others have suggested that unfavorable intranasal anatomy may increase the risk of DCR failure [8,9]. Therefore, the present study cannot determine whether recurrence in patients with a narrow nasal cavity was directly attributable to the absence of septoplasty.

There were many studies that combined endonasal procedures with DCR surgery [15,16,17]. Figueira et al. [16] reported that in 576 En-DCR cases, 81 cases (14%) needed to be combined endonasal procedures, and there was no significance in the anatomical and functional success of En-DCR. Ali et al. [15] also reported 269 En-DCR cases, demonstrating that septoplasty and middle turbinoplasty can be helpful for access to the DCR site during surgery. A previous study also reported that there was no statistical significance in combining septoplasty with En-DCR, but septoplasty can be helpful for facilitating the main surgery and reducing intranasal complications [16]. To the best of our knowledge, there was no study that reported combining septoplasty with revision En-DCR. Our study may provide clinical guidance when considering revision En-DCR in patients with recurrent symptoms and unfavorable nasal anatomy.

Surgical technique may also influence outcomes in anatomically complex revision cases. Although an endoilluminator-guided technique was used in the present study, retrograde endoscopic approaches have been introduced to facilitate identification and exposure of the lacrimal sac in selected difficult cases [18]. Because all procedures in the present study were performed using the same surgical technique, the potential impact of alternative approaches on surgical outcomes could not be evaluated.

This study is subject to several limitations. First, the small number of revision cases may have obscured the actual impact of concomitant septoplasty on revision En-DCR outcomes. Second, because septoplasty was preferentially performed in patients with marked septal deviation or a narrow nasal cavity, selection bias cannot be excluded. Therefore, the low recurrence rate in the septoplasty group should not be interpreted as a therapeutic effect. Nevertheless, the outcome in this anatomically challenging group suggests that septoplasty may help improve surgical access. Third, anesthesia type was also determined according to surgical complexity, intranasal accessibility, the need for concomitant septoplasty, and patient tolerance, rather than by random assignment. Therefore, the imbalance in anesthesia type between groups should be considered a potential confounding factor when interpreting the surgical outcomes. Fourth, as a single-center study conducted in an Asian population, the generalizability of our findings to a worldwide population is inherently limited. Furthermore, outcome assessment was limited by the lack of standardized functional and anatomical grading. Postoperative symptoms were assessed clinically rather than using a validated epiphora score or symptom questionnaire, and endoscopic ostium findings were not quantified using a standardized grading system [19,20]. Future prospective studies should incorporate both symptom-based outcome measures and objective endoscopic ostium grading.

Despite these limitations, a key strength of our study is that all patients were operated on by the same surgeon and within the same controlled environment. This consistent approach significantly reduced the variability commonly associated with differences in surgical techniques and environmental conditions.

In conclusion, revision En-DCR with concomitant septoplasty showed acceptable outcomes in selected patients with unfavorable nasal anatomy. In addition, patients who experience recurrence of symptoms after primary En-DCR and have unfavorable nasal anatomy may benefit from a multidisciplinary approach involving both ophthalmologists and otorhinolaryngologists during management. However, considering the retrospective design of this study, selective use of septoplasty, limited sample size, and lack of statistical significance in outcome differences, the present study should be interpreted cautiously. Further studies are needed to evaluate the clinical advantage of septoplasty by comparing patients with similar degrees of septal deviation.

## 5. Conclusions

Revision En-DCR with concomitant septoplasty showed acceptable outcomes in selected patients with unfavorable nasal anatomy. Septoplasty may be helpful for improving surgical access in revision cases with a narrow nasal cavity or septal deviation, although its independent benefit could not be confirmed in this study. Larger studies with more balanced patient groups are needed to better define the role of septoplasty in revision En-DCR.

## Figures and Tables

**Table 1:** Demographic data for the subjects.

Characteristics	Value
Sex	
Male	114 (29.9)
Female	267 (70.1)
Age (years)	60.18 ± 14.38
Operated side	
Right	253 (51.7)
Left	236 (48.3)
Lacrimal symptom	
Epiphora	310 (81.4)
Discharge	39 (10.2)
Dacryocystitis	32 (8.4)

Data are presented as *n* (%) or mean ± standard deviation. Sex, age, and presenting symptoms are patient-based variables (*n* = 381), whereas the operated side is a case-based variable (*n* = 489).

**Table 2:** Demographic data for the revision En-DCR subjects.

Variables	Revision En-DCR + Septoplasty	Revision En-DCR Only	*p*-Value
Number of patients	12	24	
Number of cases	15	27	
Sex			0.443
Male	4 (33.3)	5 (20.8)	
Female	8 (66.7)	19 (79.2)	
Age	56.50 ± 9.30	57.83 ± 9.90	0.700 ^†^
Operated side			0.495 ^‡^
Right	6 (40.0)	8 (29.6)	
Left	9 (60.0)	19 (70.4)	
Anesthesia for revision surgery			0.002 *
General	12 (100.0)	11 (45.8)	
Local	0 (0.0)	13 (54.2)	
DCG findings before first DCR			0.484 ^§^
Common canalicular obstruction	4 (26.7)	8 (29.6)	
Common canalicular stenosis	1 (6.7)	1 (3.7)	
NLD obstruction	5 (33.3)	14 (51.9)	
NLD stenosis	4 (26.7)	4 (14.8)	
Functional NLD obstruction	1 (6.7)	0 (0.0)	

Data are presented as *n* (%) or mean ± standard deviation. Sex, age, and anesthesia for revision surgery are patient-based variables, whereas operated side and dacryocystography (DCG) findings are case-based variables. En-DCR, endoscopic dacryocystorhinostomy; DCR, dacryocystorhinostomy; NLD, nasolacrimal duct. * Fisher’s exact test. ^†^ Independent sample *t*-test. ^‡^ χ^2^ test. ^§^ Fisher-Freeman-Halton exact test.

**Table 3:** Intraoperative and failure-related findings in revision En-DCR cases.

Variables	Revision En-DCR + Septoplasty	Revision En-DCR Only	*p*-Value
Number of cases	15	27	
Narrow nasal cavity	14 (93.3)	11 (40.7)	0.001 *
Bleeding	3 (20.0)	1 (3.7)	0.122 *
Common canalicular obstruction	1 (6.7)	5 (18.5)	0.395 *
Small bony opening	5 (33.3)	9 (33.3)	1.000 *
Ostium obstruction/stenosis	14 (93.3)	24 (88.9)	1.000 *
Scar or granulation tissue	8 (53.3)	11 (40.7)	0.525 *
Synechiae/cicatricial adhesion	10 (66.7)	8 (29.6)	0.027 *

Data are presented as *n* (%). En-DCR, endoscopic dacryocystorhinostomy. * Fisher’s exact test.

**Table 4:** Outcome of revision operation in recurrent En-DCR patients.

Variables	Revision En-DCR + Septoplasty	Revision En-DCR Only	*p*-Value
Duration between first En-DCR and revision En-DCR (months)	5 (4–12)	4 (4–12)	0.926 ^†^
Recurrence	1 (6.7)	6 (22.2)	0.390 *
Re-operation	1 (6.7)	3 (11.1)	1.000 *

Data are presented as median (interquartile range) or *n* (%). En-DCR, endoscopic dacryocystorhinostomy. * Fisher’s exact test. ^†^ Mann–Whitney U test.

## Data Availability

The data that support the findings of this study are available on request from the corresponding author.

## Abbreviations

The following abbreviations are used in this manuscript:

En-DCR	Endoscopic dacryocystorhinostomy
DCR	Dacryocystorhinostomy
NLD	Nasolacrimal duct
DCG	Dacryocystography
